# Development of oligonucleotide microarray for accurate and simultaneous detection of avian respiratory viral diseases

**DOI:** 10.1186/s12917-019-1985-7

**Published:** 2019-07-19

**Authors:** Qian Xiao, Liping Yan, Lu Yao, Jing Lei, Zhenwei Bi, Jianhua Hu, Yuqing Chen, An Fang, Hui Li, Yuan Li, Yan Yan, Jiyong Zhou

**Affiliations:** 1 MOE Joint International Research Laboratory of Animal Health and Food Safety, Institute of Immunology, Nanjing Agricultural University, Nanjing, 210095 People’s Republic of China; 2 Jiangsu Engineering Laboratory of Animal Immunology, Institute of Immunology, Nanjing Agricultural University, Nanjing, 210095 People’s Republic of China; 3 Jiangsu Detection Center of Terrestrial Wildlife Disease, College of Veterinary Medicine, Nanjing Agricultural University, Nanjing, 210095 People’s Republic of China; 4 Key Laboratory of Animal Virology, Ministry of Agriculture, Zhejiang University, Hangzhou, 310058 People’s Republic of China; 5 Collaborative Innovation Center for Diagnosis and Treatment of Infectious Diseases, The First Affiliated Hospital, Zhejiang University, Hangzhou, 310058 People’s Republic of China

**Keywords:** Avian influenza virus, Newcastle disease virus, Infectious bronchitis virus, Oligonucleotide microarray

## Abstract

**Background:**

Avian influenza virus (AIV), infectious bronchitis virus (IBV), and Newcastle disease virus (NDV) are important avian pathogens that can cause enormous economic loss on the poultry industry. Different respiratory etiological agents may induce similar clinical signs that make differential diagnosis difficult. Importantly, AIV brings about severe threat to human public health. Therefore, a novel method that can distinguish these viruses quickly and simultaneously is urgently needed.

**Results:**

In this study, an oligonucleotide microarray system was developed. AIV, including H5, H7, and H9 subtypes; NDV; and IBV were simultaneously detected and differentiated on a microarray. Three probes specific for AIV, NDV, and IBV, as well as three other probes for differentiating H5, H7, and H9 of AIV, were first designed and jet-printed to predetermined locations of initiator-integrated poly(dimethylsiloxane) for the synchronous detection of the six pathogens. The marked multiplex reverse transcription polymerase chain reaction (PCR) products were hybridized with the specific probes, and the results of hybridization were read directly with the naked eyes. No cross-reaction was observed with 10 other subtypes of AIV and infectious bursal disease virus, indicating that the oligonucleotide microarray assay was highly specific. The sensitivity of the method was at least 100 times higher than that of the conventional PCR, and the detection limit of NDV, AIV, H5, H7, and H9 can reach 0.1 EID_50_ (50% egg infective dose), except that of IBV, which was 1 EID_50_ per reaction. In the validation of 93 field samples, AIV, IBV, and NDV were detected in 53 (56.99%) samples by oligonucleotide microarray and virus isolation and in 50 (53.76%) samples by conventional PCR.

**Conclusions:**

We have successfully developed an approach to differentiate AIV, NDV, IBV, H5, H7, and H9 subtypes of AIV using oligonucleotide microarray. The microarray is an accurate, high-throughput, and relatively simple method for the rapid detection of avian respiratory viral diseases. It can be used for the epidemiological surveillance and diagnosis of AIV, IBV, and NDV.

**Electronic supplementary material:**

The online version of this article (10.1186/s12917-019-1985-7) contains supplementary material, which is available to authorized users.

## Background

At present, the demand for broiler meat products is increasing worldwide; however, avian viruses cause respiratory infections and lead to mortality in poultry flocks, which impose negative influence on chicken products [1]. The avian influenza virus (AIV), Newcastle disease virus (NDV), and infectious bronchitis virus (IBV) can cause enormous economic loss in the poultry industry worldwide. AIV belongs to the *Orthomyxovir*idae family, *Influenza A virus* genus. The AIV genome is composed of eight segments of negative-sense single-stranded RNA and classified into different subtypes according to two surface antigens: hemagglutinin (HA) and neuraminidase (NA); heretofore, there are 18 known HA and 11 known NA subtypes [2]. Among all known HA subtypes, the highly pathogenic avian influenza viruses have been restricted to H5 and H7, which not only cause considerable economic losses in the poultry industry [3] but also seriously threaten human health [4]. Other subtypes cause a milder respiratory disease, and they are designated as low pathogenicity avian influenza (LPAI) viruses. Although H9 is an LPAI, it is the dominant circulating subtype, showing a high prevalence [5]. NDV belongs to the *Paramyxoviridae* family, *Avulavirus* genus, and is an RNA-containing virus. It appears to be a sporadic epizootic disease despite vaccination programs [6]. In industrial poultry farms, birds infected with NDV must be immediately sacrificed due to the threat of infection dissemination across countries. IBV belongs to the family *Coronaviridae* and the genus *Coronavirus*. It infects chickens of all ages and causes lesions in respiratory and urogenital organs [7]. Although the IBV vaccine plays a vital role in controlling IB, IBV outbreaks frequently occur and are still considered a global epidemic [8].

These viruses, in association with bacterial agents or independently, can lead to diseases [9, 10]. In addition, the clinical symptoms caused by single- or multiple-viruses are similar, which makes it difficult for veterinarians to distinguish these viruses in the field. Therefore, sensitive and rapid detection techniques that can distinguish these respiratory viral infections are needed for the surveillance of the emergence of new viruses, outbreak management, and disease control.

Currently, virus isolation combined with hemagglutination inhibition and NA inhibition tests is considered to be the conventional gold standard; however, it is time consuming (it needs four to 6 days), has low sample throughput, and is relatively insensitive [11]. Polymerase chain reaction (PCR) is widely used to identify the aforementioned viruses via molecular diagnostic assays, and reverse transcription PCR (RT-PCR) is now becoming accepted as a new gold standard in many studies because it shows superior sensitivity [12, 13]. The real-time PCR method, which can quantitate samples, is also used; however, it requires expensive equipment [14]. Moreover, most of the aforementioned methods are used for testing only one agent in a specimen [15]. Multiplex RT-PCR assays that can involve simultaneous amplification of more than one infectious agent are also available, the advantages of the multiplex RT-PCR combine the sensitivity and speed of PCR and eliminate the need for testing clinical samples for each virus separately [16, 17]. Many studies have also been performed using multiplex real-time RT-PCR to differentiate single or mixed avian virus infection, such as NDV, AIV, and IBV [18–21]. In recent years, microarray technology has also been developed [22, 23].

The correct differentiation between viral infections is very important for accurately monitoring and effectively controlling respiratory avian diseases. Herein, we developed a rapid, concurrent, and high-throughput oligonucleotide microarray approach for the detection of avian respiratory pathogens, such as AIV, NDV, and IBV single- and mixed-virus infections, including the H5, H7, and H9 subtypes of AIV.

## Methods

### Plasmids, viruses and clinical specimens

The standard plasmids (pMD18-T-AIV-M, pMD18-T-H5, pMD18-T-H7, pMD18-T-H9, pMD18-T-NDV-F, pMD18-T-IBV-N) were obtained by our laboratory. The virus strains used in this study, including AIV, NDV, IBV, and IBDV, were obtained from the Key Animal Virology Laboratories of the Ministry of Agriculture of China and were preserved at − 80 °C (Table 1).

**Table 1:** Viruses used in the work

No	Name of isolate	Taxonomy of virus	Strain description	HA titer	Highly similar sequences^a^ (≥99%)
Avian influenza virus					
1	AIV-H1N1 P2009	Genus-*Influenza virus A*, Family-*Orthomyxoviridae*	virulent	2^9^	MH061695.1
2	AIV-H2N2 21103		virulent	2^5^	L11134.1
3	AIV-H3N8 11102		virulent	2^8^	CY005816.1
4	AIV-H4N6 20411		virulent	2^7^	GU052381.1
5	AIV-H5N1 060315		virulent	2^5^	JX565019.1
6	AIV-H6N5 20411		virulent	2^7^	CY014656.1
7	AIV-H7N3 201369		virulent	2^7^	JQ906576.1
8	AIV-H8N4 20413		virulent	2^9^	CY014659.1
9	AIV-H9N2 201313		virulent	2^9^	KF059279.1
10	AIV-H10N7 20410		virulent	2^7^	CY014671.1
11	AIV-H11N9 21103		virulent	2^9^	CY014687.1
12	AIV-H12N5 11103		virulent	2^7^	GU052216.1
13	AIV-H13N6 11103		virulent	2^6^	CY014694.1
Newcastle disease virus					
14	NDV -LaSota	Genus- *Avulavirus*, Family-*Paramyxoviridae*	vaccinal	2^8^	DQ195265.1
Infectious bronchitis virus					
15	IBV-J (F8)050309	Genus-*Coronavirus*, Family-*Coronaviridae*	virulent	–	FJ849834.1
Infectious bursal disease virus					
16	IBDV-NB(F7)	Genus-*Avibirnavirus*, Family-*Birnaviridae*	virulent	–	AY319768.2

^a^ Accession number from the GenBank databases

A total of 93 samples, including oropharyngeal and cloacal swabs, were collected from the live poultry markets of the Jiangsu Province, China. The oropharyngeal swab or cloacal swab was dipped respectively in 2 mL phosphate-buffered saline to release the fecal or tracheal materials from the swabs. Each specimen was separated into two halves: one half by extracting viral RNA was used to perform microarray and reverse transcription of cDNA for traditional PCR, while the other half was used for virus isolation. Virus isolation of these clinical samples were isolated using specific-pathogen-free embryonated hen’s eggs (Tianbang Biotechnology, China), then the type and subtype were identified by reverse transcription and conventional PCR assay, respectively [24, 25].

### Primers and probes

Sequences of virus nucleotides were downloaded from the National Center for Biotechnology Information GenBank (See Additional file 2: Table S1). The common probes and primers targeting AIV, NDV, and IBV were designed from the conserved sequences of AIV-M gene, NDV-F gene, and IBV-N gene, respectively. The probes and primers of AIV H5, H7, and H9 subtypes were based on conserved sequences of HA gene. The probes of H7 and H9 subtypes and the primers of H5, H7, and H9 subtypes have already been designed [24].

The selected nucleotide sequences were aligned by using Lasergene (DNASTAR Inc., USA), and these primers were designed using Primer Express 3.0 (Applied Biosystems, USA). All the designs for forward primers were labeled with biotin on the 5′ end. The primers and probes are listed in Table 2.

**Table 2:** Primers and probes

Name	Sequence(5′-3′)	Targeted gene and virus type	Length
H9 probe	TTCGACTGTCGCCTCATCTCTTG	Haemagglutinin gene of AIV subtype H9	157
H9-F	Biotin-CAGAACAAGAAGGCAGCAA		
H9-R	AATGTGATGACCARTGCATGG		
H7 probe	GGTTTAGCTTCGGGGCATCATG	Haemagglutinin gene of AIV subtype H7	105
H7-F	Biotin-CCATTRCAATGGCTAGAAG		
H7-R	AATAGAATACAGATWGACCCAGT		
H5 probe	GCCTCAAACTGAGTGTTCATTTTGT	Haemagglutinin gene of AIV subtype H5	210
H5-F	Biotin-GTACCACCATAGCAATGAGCAG		
H5-R	AGTCCAGACATCTAGGAATCCGT		
M probe	TCGGCTTTGAGGGGGCCTGA	M gene of all subtypes of AIV	163
M-F	Biotin-ATGAGYCTTCTRACCGAGGTCG		
M-R	GAGGTGACAGGATTGGTCTTGTC		
IBV probe	CGCCCATCCTTAATACCTTCCTCA	N gene of IBV and all pathotypes of IBV	175
IBV-F	Biotin-GTARGGAGGGNAATTTTGGTGATGA		
IBV-R	ACACACTSRTCACAAATYTTYACATAATTA		
NDV probe	GAGGTGTCAAGYTCTTCTATCACAGAACC	F gene of NDV and all pathotypes of NDV	107
NDV-F	Biotin-GTCCCRAARGTRGTGACACA		
NDV-R	GGGAAYTGTCACTATYCTDGTACA		

### RNA extraction and cDNA synthesis

The viral RNAs of AIV, NDV, and IBV strains were extracted by using the RNeasy mini kit (Qiagen Inc., CA) according to the manufacturer’s instructions. The concentration and purity of the extracted total RNA were determined by measuring the absorbance ratio at a wavelength of 260 nm over 280 nm using a NanoDrop 2000c spectrophotometer (Thermo Scientific, USA). The final extracted pellets were stored at − 80 °C.

First-strand cDNA was synthesized using AMV reverse transcriptase (TaKaRa Biotechnology, China). Briefly, the following reagents were added and mixed: 4 μL of 5× reverse transcriptase buffer, 2 μL of dNTP mixture (10 mmol/L), 1 μL of random primer (50 mmol/L), 2 μL of AMV reverse transcriptase, 0.5 μL of RNAase inhibitor (40 U/μL), 5 μL of RNA, and 5.5 μL of RNAase-free water. The reaction mixture was sequentially incubated at 42 °C for 60 min and then at 72 °C for 15 min. The cDNA was subsequently stored at − 20 °C.

### Microarray printing

Microarray was prepared in a 100,000 grade clean room. Probes were diluted with printing buffer (0.3 M phosphate buffer, 0.2% glycerin, 0.01% Triton X-100, and 1.5% mannitol) for further printing. Each dilution of probes was printed on initiator-integrated poly(dimethylsiloxane) (iPDMS), a novel solid supporting material. The oligonucleotide microarray was completed using a contact printer SmartArrayer 48 (CapitalBio, China) with approximately 0.6 nL of printing solution for each sample. Each well has positive control with biotin and negative control with printing buffer.

### Multiplex RT-PCR

The multiplex RT-PCR was conducted in a 20.0 μL reaction system with a PCR machine (Eppendorf, Germany). Multiplex RT-PCR primers were adapted as previously described for AIV, NDV, and IBV (Table 2). Multiplex RT-PCR was performed using HiScript® II One Step qRT-PCR Probe Kit (Vazyme, USA). The reaction mixture contained 2.0 μL mixed primers (IBV-F, IBV-R, NDV-F, NDV-R, M-F, M-R, H5-F, H5-R, H7-F, H7-R, H9-F, H9-R), 10.0 μL of 2× One-Step Q Probe Mix, 1.0 μL One-Step Q Probe Enzyme Mix, 6.0 μL of RNase-free water, and 1.0 μL of extracted RNA (total: 20.0 μL). The multiplex RT-PCR program was as follows: a reverse transcription step at 50 °C for 5 min, initial denaturation at 95 °C for 2 min, and finally 40 amplification cycles of 95 °C for 10 s and 54 °C for 10 s.

### Hybridization reaction

The multiplex RT-PCR product was diluted 1:4 with 2× saline sodium citrate (SSC) + 0.1% sodium dodecyl sulfate (SDS) (wash A). The diluting product was denatured in boiling water bath for 5 min and immediately cooled in an ice bath for 2 min. The microarray was preheated at 37 °C for 5 min. Then, 60 μL of the diluting product was added to the microarray chamber, incubated at 47 °C, stirred at 200 rpm for 20 min, and washed twice with 0.5× SSC + 0.2% SDS (wash B, the preheating maintenance temperature of wash B must be equal to 47 °C). Streptavidin–horseradish peroxidase (streptavidin-HRP) was diluted 1:500 with wash A. A dilute solution of streptavidin-HRP should be preheated to 47 °C, and 60 μL should be added to the microarray chamber. The oligonucleotide microarray was incubated on a shaker plate at 47 °C for 20 min at 200 rpm, washed three times with wash B, and then washed twice with 0.05 M sodium citrate buffer (wash C, room temperature). A total of 100 μL tetramethylbenzidine chromogenic reagent was added onto the microarray and was allowed to stand for 3 min to read the results with the naked eyes.

### Conventional PCR and real-time PCR

A total of 20 μL of conventional PCR mixture included 10.0 μL of 2× Taq Master Mix (Vazyme, USA), 0.2 μM of each pair of primers (Table 2), 1.0 μL of template and ddH_2_O. The standard plasmids (pMD18-T-AIV-M, pMD18-T-H5, pMD18-T-H7, pMD18-T-H9, pMD18-T-NDV-F, pMD18-T-IBV-N) served as a positive control, while ddH_2_O played the role of the negative control. The thermocycling parameters were as follows: 95 °C for 5 min, 40 cycles of 95 °C for 30 s, 54 °C for 30 s, and 72 °C for 1 min, followed by 72 °C for 10 min at the end of the reaction. PCR product was separated on a 1.0% agarose gel. The assay was repeated at least 2 times and two technical replicates were run for each assay.

The real-time PCR was performed in a 20.0 μL reaction system with a LightCycler 96 real-time PCR system (Roche, Switzerland). The 20.0 μL reaction mixture comprised 10.0 μL of SYBR Premix Ex Taq II (TaKaRa Biotechnology, China), 2.0 μL of each pair of primers (Table 2), 2.0 μL of template, and 6.0 μL of ddH_2_O. The real-time PCR program was as follows: predenaturation at 95 °C for 30 s and 40 amplification cycles of 95 °C for 5 s and 54 °C for 30 s. The positive and negative controls (pMD18-T-AIV-M, pMD18-T-H5, pMD18-T-H7, pMD18-T-H9, pMD18-T-NDV-F, pMD18-T-IBV-N and ddH_2_O) of real-time PCR were set. Fluorescent signals were obtained once per cycle upon the completion of the extension step. Samples exhibiting a cycle threshold (Ct) value of less than 35 were considered positive. The assay was repeated at least 2 times and two technical replicates were run for each assay.

### Specificity and sensitivity of the oligonucleotide microarray

The primers and probes were evaluated by using the BLAST tool, and the specificity of the oligonucleotide microarray was evaluated by cross-reactivity with other subtypes (H1, H2, H3, H4, H6, H8, H10, H11, H12, H13 AIVs) of influenza A virus and IBDV. The sensitivity of the method was evaluated using tenfold serial dilution of virus quantified by 50% egg infective dose (EID_50_). The final concentration of virus RNA was between 10^5^ and 10^− 2^ EID_50_ per reaction mixture. Each dilution of virus and negative control was tested by conventional PCR, oligonucleotide microarray, and real-time PCR.

### Reproducibility of the oligonucleotide microarray assay

To evaluate the reproducibility of the oligonucleotide microarray assay, equal amounts of five virus strains (H5, H7, H9, IBV, and NDV) were mixed, and the titer of each virus was 10^6^ EID_50_ per reaction. The mixture was tested in three independent reactions over separate days.

### Application of the oligonucleotide microarray assay

Before detecting the specimens, the co-infection models, which included various arrangements, were designed and tested to determine the detection efficiency of the oligonucleotide microarray.

The 93 clinical oropharyngeal and swabs were assayed to evaluate the feasibility of the methods for detection of AIV; IBV; NDV; and H5, H7, and H9 subtypes.

## Results

### Multiplex RT-PCR

The study developed a multiplex RT-PCR with six pairs of primers for the fragment of NDV-F, IBV-N, AIV-M, AIV-H5, AIV-H7, and AIV-H9 prior to the microarray test. The PCR product was 163 bp (AIV-M), 210 bp (AIV-H5), 105 bp (AIV-H7), 157 bp (AIV-H9), 107 bp (NDV), and 175 bp (IBV) (See Additional file 1: Figure S1).

### Oligonucleotide microarray assay

**Fig. 1:**
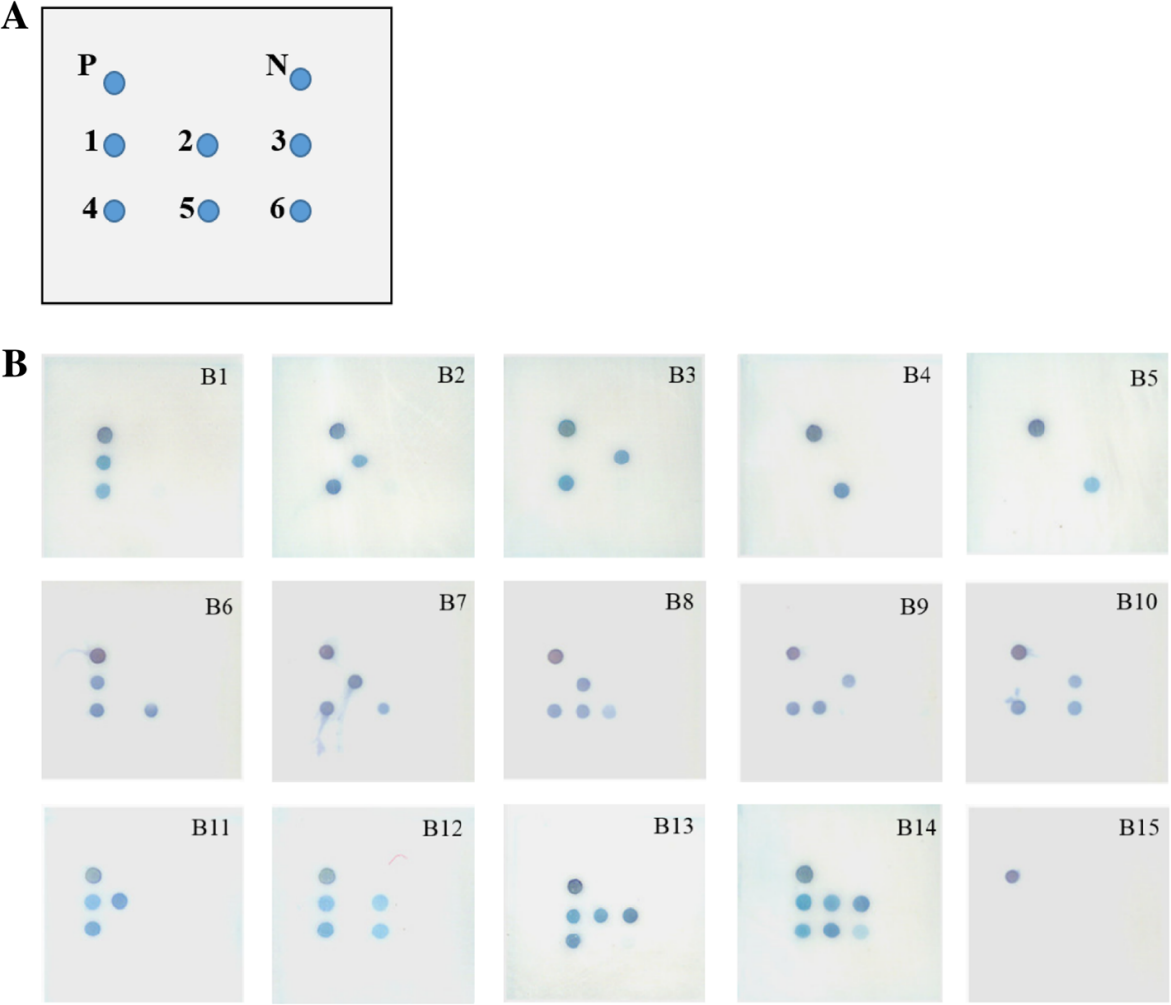
Detection and typing of NDV, IBV, or AIV using oligonucleotide microarrays. **a** Microarray map. Each dot indicates the spotted position of each probe. P: Positive control; N: Negative control; 1: AIV-H5; 2: AIV-H7; 3: AIV-H9; 4: AIV-M; 5: IBV-N; 6: NDV-F. **b** Detection and typing results shown on the microarrays. B1: H5 AIV; B2: H7 AIV; B3: H9AIV; B4: IBV; B5: NDV; B6: H5 AIV + NDV; B7: H7 AIV + NDV; B8: H7 AIV + IBV + NDV; B9: H9 AIV + IBV; B10: H9 AIV + NDV; B11: H5 AIV + H7 AIV; B12: H5 AIV + H9 AIV + NDV; B13: H5 AIV + H7 AIV + H9 AIV; B14: H5 AIV + H7 AIV + H9 AIV + IBV + NDV; B15: Negative control

A single virus or various combinations of the viruses (AIV, NDV, and IBV) were tested using the oligonucleotide microarray assay following the multiplex RT-PCR. All viruses were detected and typed expressly, and no cross-reaction with other probes was found (Fig. 1). In this study, the results were clearly recognizable with the naked eyes, and no accessional imaging equipment was needed. This finding indicated that the simultaneous detection, differentiation, and typing of AIV, NDV, and IBV can be inexpensively and easily achieved using the oligonucleotide microarray.

### Specificity and sensitivity of the oligonucleotide microarray assay

**Fig. 2:**
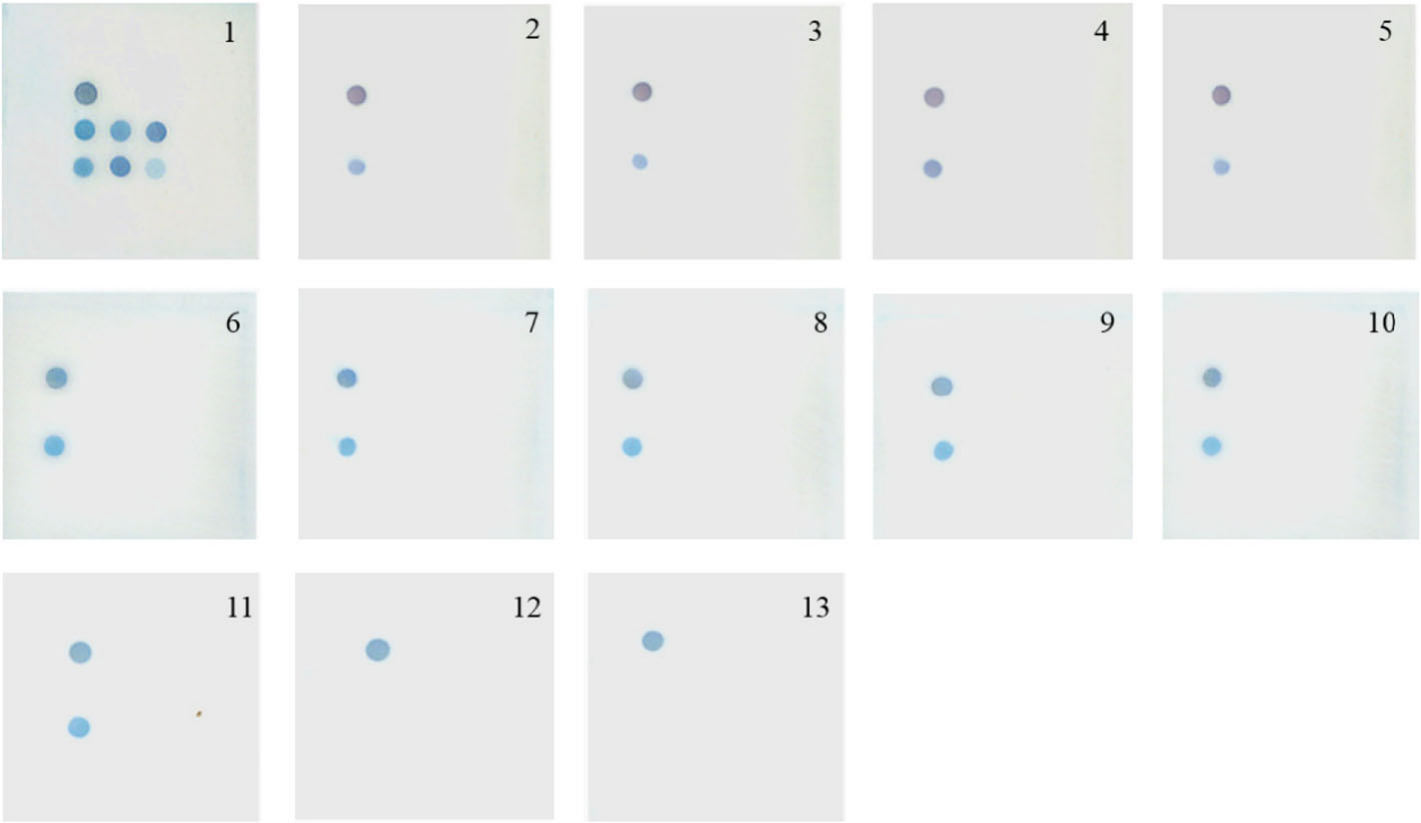
Specificity of oligonucleotide microarrays. Rapid detection of the other avian respiratory viruses and H1–H13 AIVs using oligonucleotide microarrays. 1: Positive control; 2: H1 AIV; 3: H2 AIV; 4: H3 AIV; 5: H4 AIV; 6: H6 AIV; 7: H8 AIV; 8: H10 AIV; 9: H11 AIV; 10: H12 AIV; 11: H13 AIV; 12: IBDV; 13: Negative control

The oligonucleotide array had good specificity, and no cross-reactivity with any of the other avian respiratory viruses was found. The oligonucleotide microarray assay was examined by testing H1–H13 (except H5, H7, and H9) AIVs and IBDV. The influenza viruses could react with the M probe only (Fig. 2).

The detection limit comparison test among conventional PCR assay, oligonucleotide microarray, and the real-time PCR was performed. The results showed that the conventional PCR assay could be seen on agarose gel when infectious virus titer was 10^2^ EID_50_ per reaction, except for NDV, which showed positive results only when the virus titer was more than 10^3^ EID_50_ per reaction (Table 3). The detection limit of real-time PCR was 0.1 EID_50_ per reaction with the H5 and NDV, and others were approximately 1 EID_50_ per reaction (Table 3). Moreover, the sensitivity of the microarray relative to the detectable infectious virus titer was 0.1 EID_50_ per reaction with the NDV, M, H5, H7, and H9. The detection limit of IBV could reach 1 EID_50_ (Table 3). This finding showed that the oligonucleotide microarray sensitivity was at least 100 times higher than that of the conventional PCR in this study, consistent with or even slightly better than real-time PCR.

**Table 3:** Sensitivity of the conventional PCR, oligonucleotide microarray, and real-time PCR

No	EID_50_	Conventional PCR								Oligonucleotide microarray								Real-time PCR							
		H5	H7	H9	H5	H7	H9	IBV	NDV	H5	H7	H9	H5	H7	H9	IBV	NDV	H5	H7	H9	H5	H7	H9	IBV	NDV
		M								M								M							
1	1 × 10^5^	+++	+++	+++	+++	+++	+++	+++	+++	+++	+++	+++	+++	+++	+++	+++	+++	+++	+++	+++	+++	+++	+++	+++	+++
2	1 × 10^4^	+++	+++	+++	**+++**	**+++**	**+++**	+++	++	**+++**	**+++**	**+++**	+++	+++	+++	+++	+++	+++	+++	+++	+++	+++	+++	+++	+++
3	1 × 10^3^	++	++	++	**++**	**++**	**++**	++	+	**+++**	**+++**	**+++**	+++	+++	+++	+++	+++	+++	+++	+++	+++	+++	+++	+++	+++
4	1 × 10^2^	+	+	+	**+**	**+**	**+**	+	–	**+++**	**+++**	**+++**	+++	+++	+++	++	+++	++	++	++	++	+++	++	++	+++
5	1 × 10^1^	–	–	–	**–**	**–**	**–**	–	–	**+++**	**+++**	**+++**	+++	+++	+++	++	+++	+	+	+	++	++	+	+	++
6	1 × 10^0^	–	–	–	**–**	**–**	**–**	–	–	**++**	**++**	**++**	++	++	++	+	++	+	+	+	+	+	–	–	+
7	1 × 10^−1^	–	–	–	–	–	–	–	–	**+**	**+**	**+**	**+**	**+**	**+**	–	**+**	–	–	–	+	–	–	–	+
8	1 × 10^−2^	–	–	–	–	–	–	–	–	–	–	–	–	–	–	–	–	–	–	–	–	–	–	–	–

Serial tenfold dilutions of virus from 10^5^ to 10^−2^ EID_50_ were tested for the conventional PCR, oligonucleotide microarray, and real-time PCR. The products of conventional PCR were separated using 1% agarose gel electrophoresis. +++ represents a very bright band/spot/signal, ++ represents a moderately bright band/spot/signal, + represents a weak band/spot/signal, and − represents no band/spot/signal

### Reproducibility of the oligonucleotide microarray assay

To evaluate the reproducibility of the oligonucleotide microarray assay, one mixture of five virus strains was tested in three independent reactions over separate days. Upon visual inspection of three independent reactions, all pathogens could be detected. The results showed that oligonucleotide microarray assay has high reliability and reproducibility.

### Co-infection models

The co-infection results proved that this method could detect mixed infection accurately, whether it was AIV, IBV, and NDV triplex infection or between each of the multiplex infections. In the mixture proportion of each component at the same level and in the gap, the method could accurately detect all the viruses in the co-infection system (Table 4).

**Table 4:** Detection of the co-infection models by oligonucleotide microarray

Virus mixture	M	H5	H7	H9	IBV	NDV
10^4^ H5 + 10^3^ IBV+ 10^4^ NDV	+	+	–	–	+	+
10^3^ H7 + 10^2^ IBV+ 10^3^ NDV	+	–	+	–	+	+
10^3^ H9 + 10^1^ IBV	+	–	–	+	+	–
10^2^ IBV + 10^2^ NDV	–	–	–	–	+	+
10^3^ H5 + 10^2^ H7 + 10^2^ IBV	+	+	+	–	+	–
10^2^ H7 + 10^2^ H9 + 10^1^ NDV	+	–	+	+	–	+
10^1^ H7 + 10^1^ NDV	+	–	+	–	–	+
10^1^ H5 + 10^1^ H7 + 10^1^ H9	+	+	+	+	–	–
10^0^ H5 + 10^1^ H7 + 10^0^ H9 + 10^0^ IBV	+	+	+	+	+	–
10^0^ H5 + 10^0^ H7 + 10^0^ H9 + 10^0^ NDV + 10^0^ IBV	+	+	+	+	+	+

### Clinical specimen detection, diagnostic sensitivity, and diagnostic specificity

A total of 93 field samples were analyzed using the oligonucleotide microarray, conventional PCR, and virus isolation assay. The results showed that the respective positive detection rates with conventional PCR, oligonucleotide microarray, and the virus isolation were 30.11, 33.33, and 33.33% for AIV and 23.66% for NDV (Table 5). For AIV, a total of 31 positive samples among 93 clinical samples were detected with oligonucleotide microarray and virus isolation, while 28 positive samples were detected with conventional PCR. For NDV, 22 positive samples were detected, and no obvious difference was found for the identification of these methods. None of the field samples was IBV positive. Oligonucleotide microarray and virus isolation showed higher detection rates of H5, H7, and H9 than conventional PCR. However, these methods had the same detection rate for NDV and IBV. Moreover, using the microarray method, two samples were found to be co-infected with H7 and H9 AIVs, and no other co-infected samples were observed in the study. The results of AIV, NDV, and IBV detection in the specimens by different methods were compared and showed that microarray detection could be applied for laboratory surveillance and diagnosis of the pathogens in clinical fields.

**Table 5:** Virus detection in field samples by conventional PCR, oligonucleotide microarray and virus isolation

Virus	Conventional PCR	Oligonucleotide microarray	Virus isolation^a^
	Positive	Positive	Positive^b^
Influenza A	28/93 (30.11%)	31/93 (33.33%)	31/93 (33.33%)
H5	16/93 (17.20%)	18/93 (19.35%)	18/93 (19.35%)
H7	6/93 (6.45%)	7/93 (7.53%)	9/93 (9.68%)
H9	3/93 (3.23%)	3/93 (3.23%)	5/93 (5.38%)
H5, H7	0/93 (0%)	0/93 (0%)	0/93 (0%)
H7, H9	0/93 (0%)	2/93 (2.15%)	0/93 (0%)
H5, H9	0/93 (0%)	0/93 (0%)	0/93 (0%)
H5, H7, H9	0/93 (0%)	0/93 (0%)	0/93 (0%)
IBV	0/93 (0%)	0/93 (0%)	0/93 (0%)
NDV	22/93 (23.66%)	22/93 (23.66%)	22/93 (23.66%)
Positive	50/93 (53.76%)	53/93 (56.99%)	53/93 (56.99%)

^a^Virus isolation was performed on specific-pathogen-free egg embryo

^b^The type and subtype were identified by reverse transcription and conventional PCR assay, respectively

Virus isolation was used as a standard method in our studies. The diagnostic sensitivity and specificity were both 100% for the oligonucleotide microarray, whereas those for the conventional PCR were 94.33 and 100%, respectively (Table 6).

**Table 6:** The diagnostic sensitivity, and diagnostic specificity of conventional PCR and oligonucleotide microarray

Target	Sensitivity ^a^(TP/(TP + FN))		Specificity ^b^ (TN/(TN + FP))	
	conventional PCR	oligonucleotide microarray	conventional PCR	oligonucleotide microarray
Total	94.33% (50/53)	100%(53/53)	100%(40/40)	100%(40/40)
AIV	90.32%(28/31)	100%(31/31)	100%(62/62)	100%(62/62)
H5	88.89%(16/18)	100%(18/18)	100%(75/75)	100%(75/75)
H7	66.67% (6/9)	100%(9/9)	100%(84/84)	100%(84/84)
H9	60% (3/5)	100%(5/5)	100%(88/88)	100%(88/88)
IBV	100% (0/0)	100%(0/0)	100%(93/93)	100%(93/93)
NDV	100% (22/22)	100%(22/22)	100%(71/71)	100%(71/71)

Virus isolation was as the gold standard, the diagnostic sensitivity, and diagnostic specificity of conventional PCR and oligonucleotide microarray for detecting viral were identified in 93 field samples. ^a^ TP, true positive; FN, false negative; Sensitivity = TP/(TP + FN) × 100%. ^b^ TN, true negative; FP, false positive; Specificity = TN/(TN + FP) × 100%

## Discussion

AIV, NDV, and IBV are highly contagious pathogens with high incidence in poultry [26, 27]. They pose a considerable threat to the poultry industry [28]. For diagnosing avian viral infection, many methods have been developed. For example, virus isolation, RT-PCR, real-time RT-PCR, fluorescent antibody test, and enzyme-linked immunosorbent assay are the currently used methods for the laboratory diagnosis of avian viruses. However, each of these methods has its advantages and limitations. The disadvantages of these methods include the following: time consuming, nonspecific, expensive, or labor-intensive [12, 29, 30]. To establish clinical diagnosis, accurate and fast multiplex detection of avian respiratory viruses is very important. In recent years, multiplex RT-PCR and microarray technology are resoundingly applied to detect AIV and its subtypes and diagnose multiple infections such as combination of NDV and AIV [31].

The diagnosis of virus types based on microarray technology can improve the quality and shorten the analysis duration in molecular diagnosis of infectious diseases. Moreover, it can satisfy the needs of simultaneous detection of multiple viruses and screening large numbers of pathology samples [32, 33]. Microarray assays for avian diseases have been previously reported. For disease diagnosis, there are oligonucleotide microarrays that can screen not only AIV and NDV but also IBV and IBDV [23]. A DNA suspension array-based assay for avian respiratory viruses, which can identify AIV, NDV, IBV, and infectious laryngotracheitis virus, has also been reported [34]. Electronic microarray assays have been used to simultaneously detect all AIV H and N subtypes and pathotyping of NDV [22].

In the present study, a microarray technology-based iPDMS with “absolute” zero background was developed, which can simplify data analysis and reduce nonspecific interactions [35, 36]. This is the first time to use this material to detect the nucleotides of avian diseases. In addition, 48 samples can be detected at once, and the results can be read visually. The microarray can simultaneously detect AIV, NDV, and IBV and accurately distinguish AIV haemagglutinin subtypes H5, H7, and H9, whereas virus isolation in embryonated eggs and conventional PCR can only detect one agent in a sample. The duration of this assay without time required for the viral RNA extraction is 2–3 h, and 48 specimens can be simultaneously assayed. We evaluated the capability of the developed assay by comparing its sensitivity with conventional PCR and real-time PCR. The virus titer was 1 EID_50_ per reaction, which can be detected by microarray. The result clearly shows that the limits of detection reached 1 EID_50_ per reaction for NDV and 0.1 EID_50_ per reaction for M, H5, H7, H9, and IBV, which was at least 100 times more sensitive than that of conventional PCR. Furthermore, the detection limit of oligonucleotide microarray is the same as real-time PCR for H5 and NDV and more sensitive than real-time PCR for IBV and H7, H9, and M of AIV. The detection results of the co-infection model also proved that the method established in this study can be used for the accurate detection of mixed infection, which, to a certain degree, also shows the viability of this method. Moreover, 93 avian field samples were used to test the effectiveness and reliability of the microarray, and the experimental results were consistent with the virus isolation. Furthermore, the co-infection situation can be detected in clinical practice by the microarray. The diagnostic sensitivity and specificity of the oligonucleotide microarray were both 100%.

In summary, the microarray assay provides an alternative high-throughput molecular diagnostic platform for susceptive and specific detection of several major viruses generally seen as vital causes of viral respiratory diseases in poultry. The microarray assay proves to be time-saving in that the entire experiment process, including PCR, took only 2.5 h. Compared with other methods, the microarray assay is more efficient because it can detect three viruses and identify HA subtypes of AIV. It is also convenient because the results of the experiments can be determined with the naked eyes, disregarding the need for expensive experimental apparatus and complicated analysis.

## Conclusions

The oligonucleotide microarray with high sensitivity and specificity was successfully developed for the rapid detection of AIV, NDV, and IBV. More importantly, it can simultaneously identify H5, H7, and H9 subtypes of AIV. This assay will be a useful tool in the control and management of AIV, NDV, and IBV.

## Additional files

Additional file 1: **Figure S1.** PCR products of F (NDV), N (IBV), M (AIV), H9 (AIV), H7 (AIV) and H5 (AIV) gene fragments. (DOCX 235 kb)

Additional file 2: **Table S1.** Accession numbers of the GenBank reference sequences used to design the primer. (DOCX 27 kb)

## Data Availability

The dataset analyzed during the current study is available from the corresponding author on reasonable request.
