# Supplementary material for: Development of oligonucleotide microarray for accurate and simultaneous detection of avian respiratory viral diseases
Source: BMC Vet Res. 2019 Jul 19;15:253. doi: 10.1186/s12917-019-1985-7 (PMC6642548; doi:10.1186/s12917-019-1985-7)
Supplement: Supplementary file 1 — Figure S1. PCR products of F (NDV), N (IBV), M (AIV), H9 (AIV), H7 (AIV) and H5 (AIV) gene fragments. (DOCX 235 kb) [file 12917_2019_1985_MOESM1_ESM.docx]

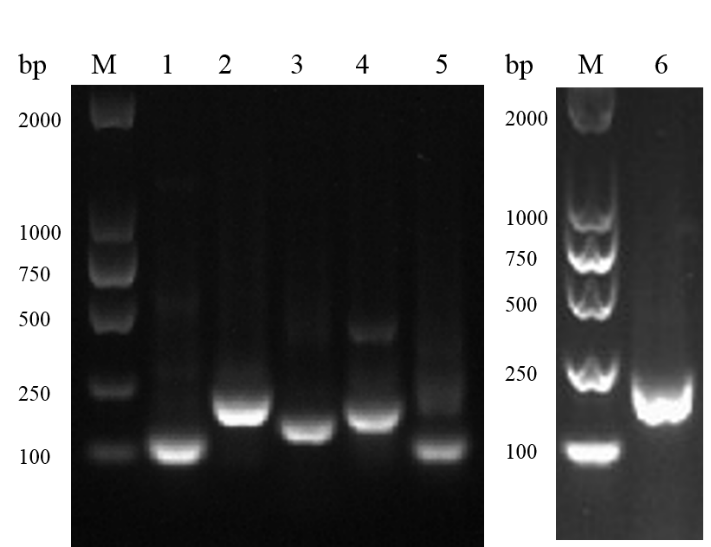


Fig. S1 PCR products of F (NDV), N (IBV), M (AIV), H9 (AIV), H7 (AIV) and H5 (AIV) gene fragments. M: 2000 bp marker (Invitrogen), 1: NDV –LaSota (NDV-F); 2: IBV-J (F8)050309 (IBV-N); 3: AIV-H9N2 201313 (AIV-M); 4: AIV-H9N2 201313 (AIV-H9); 5: AIV-H7N3 201369 (AIV-H7); 6: AIV-H5N1 060315 (AIV-H5).
