# Supplementary material for: Development of oligonucleotide microarray for accurate and simultaneous detection of avian respiratory viral diseases
Source: BMC Vet Res. 2019 Jul 19;15:253. doi: 10.1186/s12917-019-1985-7 (PMC6642548; doi:10.1186/s12917-019-1985-7)
Supplement: Supplementary file 2 — Table S1. Accession numbers of the GenBank reference sequences used to design the primer. (DOCX 27 kb) [file 12917_2019_1985_MOESM2_ESM.docx]

Table S1 Accession numbers of the GenBank reference sequences used to design the primer.

| **M genes of AIV:** | | | | |
| --- | --- | --- | --- | --- |
| HM142730.1 | HM142744.1 | KF013943.1 | KF013911.1 | KF013919.1 |
| JN605376.1 | KF013903.1 | KF874484.1 | JN605379.1 | KF259294.1 |
| CY146567.1 | EU559252.1 | KF259225.1 | KM234904.1 | JQ924788.1 |
| KF006417.1 | DQ482662.1 | HM116535.1_ | U49117.1 | GQ219713.1 |
| CY125010.1 | AY651431.1 | KF259271.1 | CY146615.1 | CY146719.1 |
| KF259255.1 | KC876694.1 | KF357790.1 | CY117196.1 | CY117374.1 |
| KF259292.1 | CY005431.1 | KM2222551.1 | JX175256.1 | KC261671.1 |
| JX286592.1 | KM222559.1 | KF357814.1 | KJ439882.1 | CY098265.1 |
| JX051232.1 | KJ439884.1 | CY005448.1 | KC709826.1 | EF597282.1 |
| KC261680.1 | CY005463.1 | KJ439889.1 | CY005454.1 | AY651428.1 |
| CY100635.1 | EU492524.1 | CY146607.1 | CY005469.1 | KF357817.1 |
| KJ439890.1 | KJ764714.1 | KJ579957.1 | KF259293.1 | KJ881014.1 |
| CY076895.1 | KC282882.1 | JX485427.1 | HM142731.1 | CY005487.1 |
| CY146551.1 | CY111609.1 | EU880343.1 | EF597301.1 | KC683706.1 |
| AY651430.1 | CY146583.1 | KM2348751.1 | DQ095633.1 | AF468843.1 |
| DQ095649.1 | DQ835771.1 | HM172132.1 | HM172457.1 | DQ835773.1 |
| GU477540.1 | FJ784874.1 | HM172123.1 | KC261482.1 | HM172124.1 |
| HM172124.1 | EF124111.1 | EU874901.1 | AY609315.1 | EF175672 |
| AY950237.1 | HM172150.1 | HM172134.1 | HM172159.1 | HM172158.1 |
| HM172136.1 | HM172153.1 | HM172135.1 | HM172118.1 | HM1721524.1 |
| AY653194.1 | EF124094.1 | EF124164.1 | AY950243.1 | GU727627.1 |
| AY950243.1 | JX570836.1 | JX570860.1 | JX565022.1 | KJ719462.1 |
| KJ174940.1 | KF150637.1 | JQ041413.1 | JQ041411.1 | CY146655.1 |
| KM234810.1 | EF634335.1 | JQ041414.1 | EF597296.1 | EF597281.1 |
| GU727656.1 | CY091630.1 | JQ973697.1 | KM540104.1 | KJ476676.1 |
| KJ476660.1 | JX304755.1 | CY109301.1 | CY109661.1 | HM144731.1 |
| EU050076.1 | KJ021051.1 | EU050040.1 | EU050064.1 | EU050008.1 |
| CY109341.1 | JF965219.1 | JQ294800.1 | KJ200702.1 | KM234776.1 |
| EU050075.1 | HM142741.1 | JF965225.1 | HM142745.1 | HM144869.1 |
| CY109509.1 | CY109229.1 | CY109253.1 | CY109645.1 | CY109317.1 |
| CY109285.1 | KJ200718.1 | KJ200854.1 | KJ200870.1 | JX297852.1 |
| JX293560.1 | HM144800.1 | HM144876.1 | HM050394.1 | HM144886.1 |
| GU220602.1 | JF965223.1 | JF965234.1 | KJ200687.1 | KJ200758.1 |
| KJ200830.1 | KJ200886.1 | CY109581.1 | CY110685.1 | CY109629.1 |
| CY146623.1 | JX304771.1 | KJ200902.1 | GU324777.1 | CY109365.1 |
| JF965218.1 | HM144879.1 | JF965248.1 | CY109261.1 | JX304763.1 |
| CY109637.1 | HM144892.1 | CY109725.1 | CY109685.1 | CY005494.1 |
| HM144895.1 | CY146911.1 | CY147031.1 | CY147071.1 | AY274263.1 |
| AY253755.1 | KM054797.1 | KM879368.1 | KM879376.1 | KM879384.1 |
| KF259442.1 | KF259238.1 | KF259279.1 | KF259243.1 | KF542878.1 |
| KF042107.1 | KF295218.1 | KF042106.1 | KF188297.1 | JQ906588.1 |
| KC876686.1 | KC899672.1 | KC162239.1 | KC464601.1 | KC821180.1 |
| EU559257.1 | KJ508893.1 | EF061125.1 | AF523482.1 | AF523499.1 |
| JF916716.1 | KP081536.1 | GQ373135.1 | DQ064407.1 | DQ997498.1 |
| **H5 genes of AIV:** | | | | |
| DQ659327.1 | AY741213.1 | EF587277.1 | EU008584.1 | FJ225472.1 |
| GQ184221.1 | EU717857.1 | EF456805.1 | EF441280.1 | EU623467.1 |
| DQ343150.1 | DQ992755.1 | AY609312.1 | DQ320876.1 | AF509017.1 |
| AY651349.1 | AF509016.1 | AY575876.1 | AM183669.1 | EF473081.1 |
| GU052150.1 | AY575877.1 | DQ914814.3 | EU874899.2 | FJ654295.1 |
| EF541412.1 | GU186708.1 | FJ842476.1 | CY029615.1 | JQ794478.1 |
| HM006733.1 | DQ095625.2 | AY623430.1 | AB189053.1 | DQ320875.1 |
| AB189061.1 | CY036173.1 | DQ992719.1 | DQ992813.1 | DQ320939.1 |
| DQ366338.1 | FJ602808.1 | AB436550.1 | GQ227391.1 | CY063318.2 |
| EF587277.1 | DQ100555.1 | DQ100554.1 | AF144305.1 | GU186708.1 |
| DQ914814.3 | AY676035.1 | CY036205.1 | CY014465.1 | EF541394.1 |
| AF036356.1 | EU122399.1 | EF619980.1 | GQ227359.1 | DQ360835.1 |
| CY029639.1 |  |  |  |  |
| **H7 genes of AIV:** | | | | |
| KR905377.1 | KF445401.1 | KM879365.1 | KP055076.1 | CY191843.1 |
| CY095570.1 | AB269694.2 | KR905371.1 | EF470586.1 | KM879381.1 |
| KF445398.1 | L37794.1 | EF470585.1 | AB269692.2 | JN966905.1 |
| AB269696.2 |  |  |  |  |
| **H9 genes of AIV:** | | | | |
| JX273542.1 | JX273553.1 | JQ609664.1 | JQ609664.1 | JX273544.1 |
| JX273543.1 | EF063731.1 | JX273546.1 | KC879299.1 | JX273545.1 |
| JX273549.1 | JX310066.1 | JX273548.1 | JX273547.1 | JX273552.1 |
| JX273560.1 | JX273557.1 | JN540058.1 | JX273563.1 | KP767371.1 |
| JX273562.1 | JX273561.1 | JX273564.1 | CY184125.1 | KP767395.1 |
| KP767391.1 | KP767382.1 | JX273566.1 | JX273565.1 | KP767397.1 |
| JX273570.1 | JX273569.1 | JX273568.1 | KP767402.1 |  |
| **N genes of IBV:** | | | | |
| AF352308.1 | FJ849832.1 | GQ149079.1 | AF352309.1 | AY363965.1 |
| AY839145.1 | AY842861.1 | DQ352152.1 | EF591036.1 | DQ352148.1 |
| FJ849835.1 | EU889032.1 | FJ430685.1 | FJ849830.1 | M85245.1 |
| AF199412.1 | AY028296.1 | AF352310.1 | AY363966.1 | DQ084440.1 |
| AY790344.1 | DQ377139.1 | AY121093.1 | AY942746.1 | AJ311317.1 |
| AY790353.1 | AY606327.2 | AY319651.1 | AY761141.1 | EU418976.1 |
| EU526388.1 | EU637854.1 | EU714028.1 | FJ888351.1 | GU393334.1 |
| HQ848267.1 |  |  |  |  |
| **F genes of NDV:** | | | | |
| FJ426563.1 | KJ621045.1 | FJ882015.1 | FJ882014.1 | AJ629063.1 |
| AF456437.1 | AF456438.1 | KJ136261.1 | KJ136258.1 | KF828884.1 |
| JQ517285.1 | FJ011448.1 | HQ997395.1 | HM625835.1 | EU315123.1 |
| EF464163.1 | EF540729.1 | KX242342.1 | KC424431.1 | KF208470.1 |
| EF128055.1 | EU330230.1 | AF456444.1 | KC424427.1 | KF055273.1 |
| AF048763.1 | DQ195265.1 | KP001163.1 | KF724899.1 | JX840455.1 |
| JQ268609.1 | FJ969394.1 | HM748948.1 | HM748944.1 | DQ485261.1 |
| AF079324.1 | KU644588.1 | AY508514.1 | KX268351.1 |  |
